# The contribution of human agricultural activities to increasing evapotranspiration is significantly greater than climate change effect over Heihe agricultural region

**DOI:** 10.1038/s41598-017-08952-5

**Published:** 2017-08-18

**Authors:** Minzhong Zou, Jun Niu, Shaozhong Kang, Xiaolin Li, Hongna Lu

**Affiliations:** Center for Agricultural Water Research in China, China Agricultural University, Beijing, 100083 China

## Abstract

Evapotranspiration (ET) is a major component linking the water, energy, and carbon cycles. Understanding changes in ET and the relative contribution rates of human activity and of climate change at the basin scale is important for sound water resources management. In this study, changes in ET in the Heihe agricultural region in northwest China during 1984–2014 were examined using remotely-sensed ET data with the Soil and Water Assessment Tool (SWAT). Correlation analysis identified the dominant factors that influence change in ET per unit area and those that influence change in total ET. Factor analysis identified the relative contribution rates of the dominant factors in each case. The results show that human activity, which includes factors for agronomy and irrigation, and climate change, including factors for precipitation and relative humidity, both contribute to increases in ET per unit area at rates of 60.93% and 28.01%, respectively. Human activity, including the same factors, and climate change, including factors for relative humidity and wind speed, contribute to increases in total ET at rates of 53.86% and 35.68%, respectively. Overall, in the Heihe agricultural region, the contribution of human agricultural activities to increased ET was significantly greater than that of climate change.

## Introduction

Evapotranspiration (ET) consists of evaporation and plant transpiration processes which move water from the earth’s surface to the atmosphere. ET is a key part of the water cycle, which is responsible for the distribution of water and energy on land surfaces, especially in arid and semi-arid areas^1, 2^. Many attempts to accurately estimate the rate of ET from different land surface covers have been made in agriculture, meteorology, hydrology, soil science and other related disciplines^3^. However, ET is greatly influenced by the spatial environment, and the heterogeneity of environmental factors makes it difficult to use traditional methods to simulate or predict regional ET^4^.

To obtain ET data at a regional scale, remote sensing methods are preferable because they offer the advantages of wide spatial coverage and fast updating. Muthuwatta *et al*.^5^ estimated a 1-year evapotranspiration distribution using the SEBS model and 19 cloud-free MODIS images, and used the water balance model to obtain total water demand. Alexandridis *et al*.^6^ used the SEBAL model with NOAA/AVHRR data and ESA Landsat TM/ETM+ remotely-sensed images to calculate the spatial distribution of daily ET on specific dates during the crop growth period, and then used the Lambda method to estimate the spatial distribution of seasonal ET. Yang *et al*.^7^ coupled an irrigation application model with the ETWatch platform to simulate the spatial distribution of ET in the Haihe Plain in northern China. Remote sensing technology is continually improving and it is now possible to combine a distributed hydrological model with data provided by remote sensing technology in order to estimate long-term ET changes over recent years. The Soil and Water Assessment Tool (SWAT) is a distributed hydrological model that has been widely used in hydrological cycle studies around the world^8^. Studies that combine remote sensing with a SWAT model to simulate regional ET are relatively rare in the SWAT literature^9^. Immerzeel and Droogers^10^ used remotely-sensed ET data extracted from a SEBAL model as the observed data to calibrate the SWAT model for the upper reaches of the Bhima basin in India. Immerzeel *et al*.^11^ subsequently used the calibrated SWAT model to predict ET and crop yield, and to analyze the water productivity in the basin. Awan *et al*.^12, 13^ used a SWAT model which had been calibrated using the remotely-sensed ET data extracted from a SEBAL model to predict the impacts of climate change on the temporal and spatial distribution of groundwater and consumptive water use in the Lower Chenab Canal of the Indus basin in Pakistan. Combining a SWAT model with remotely-sensed ET data is a relatively new method of calibrating a SWAT model, it provides a technique for estimating the long range ET in a region.

Previous studies of changes in ET in response to environmental changes have mainly considered the separate effects of two major factors: climate change and human activity. Cohen *et al*.^14^ found that in Israel the increase in ET during the period 1964–1998 was caused by increases in vapour pressure deficit and in wind speed. Qian *et al*.^15^ used the surface model CLM 3.0, combined with precipitation, temperature, solar radiation, and other observational data, and found that global terrestrial ET and precipitation changes were highly correlated. Jung *et al*.^16^ studied the trends and spatial distributions of global ET and found that the decrease in global ET since 1998 was probably due to a decrease in relative humidity. Liu *et al*.^17^ studied changes in ET per unit area in response to optimization scenarios for crop planting patterns in Qingyuan irrigation district, and found that ET per unit area decreased as the proportion of grain crops was reduced. It is difficult to separately quantify the contributions of human activity and of climate change to changes in ET because there are few ET data available and models are not easy to construct. There have been few studies in this area.

There are several methods available to quantify the effects of factors which influence crop ET, such as regression analysis, principal component analysis, factor analysis, and artificial neural network analysis. Wang *et al*.^18^ analyzed the large scale spatial distribution of crop ET using geographic information systems (GIS) data and principal component analysis. They concluded that the main influences on ET from winter wheat in northern China were thermodynamics, water, and micro-topography. Hu *et al*.^19^ used GIS data and factor analysis of 13 factors that influenced ET from spring wheat in Hexi Corridor. Their results showed that the three principal factors were meteorology, wind speed, and geography. However, multiple time series analysis would have been a better method to identify the major influences on ET in an agricultural region. Where there is a limited amount of water available for crop use, it is critical to identify the core factors that influence ET in order to study water consumption in arid conditions. However, there are few studies of ET in arid agricultural areas that use multiple time series analysis.

The agricultural region of the Heihe River basin, located in the middle of Hexi Corridor in northwest China, is important for grain, vegetable, and seed production. The basin has a key role in the water cycle and in the economic activity of the region. ET is the main consumption component (i.e. it effectively removes water from the watershed) of the water cycle on agricultural land. It can be considered as either ET per unit area or as total ET to explore the factors that have major influence on it. To be able to accurately identify the different contributions of human agricultural activities and of climate change to changes in ET is of great practical importance because to do so will enable growers to optimally use water and best respond to future climate change. The main objectives of this study are to identify the main factors that influence ET per unit area and total ET, and to calculate the contribution rates of these factors to changes in ET over a recent 31-year period. To this end, the spatiotemporal distribution of ET in this agricultural region over the past three decades was simulated by a SWAT model, and the remotely-sensed ET data was used to calibrate and validate the model simulations.

## Methods

### Study area

**Figure 1:**
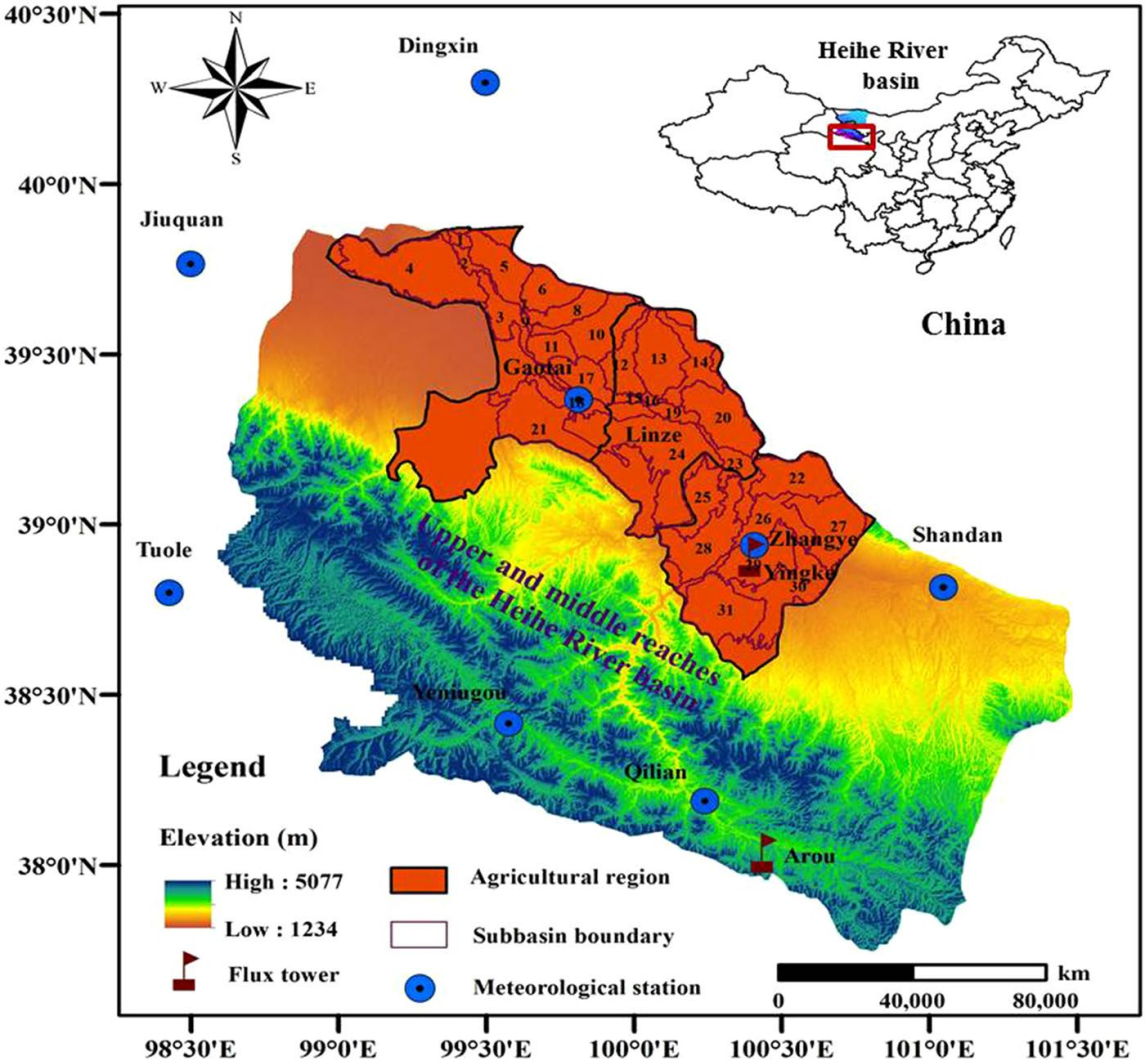
The area of study, showing the agricultural region in the Heihe River basin. The DEM of 30 m spatial resolution obtained from the Date Management Center of the Heihe Research Program (http://heihedata.org/). This figure was created using ArcGIS10.0 software provided by the Environmental Systems Research Institute (http://www.esri.com).

The Heihe River basin is the second largest inland river basin in northwest China. The agricultural region of the basin is mainly between 38°32′–39°55′N and 99°01′–100°46′E. It includes Ganzhou District, Linze County, and Gaotai County, and has a total area of 9501.85 km^2^ (Fig. 1). The average annual temperature of the agricultural region is 2.8–7.6 °C and the annual sunshine is 3000–4000 h. The average annual rainfall is 129.6 mm, mainly in the period June–September which accounts for 70–75% of the annual precipitation. Annual potential evaporation is 1400 mm^20^. Agriculture is mainly irrigated, with irrigation water drawn from Heihe River channels and local groundwater resources. The grain crops are mainly corn and spring wheat. Cash crops are mainly cotton, vegetables, oil and suchlike. Plastic film mulch is used mainly in the grain crop areas.

### 31-year retrospective ET data generation

The 31-year ET data, which cover the middle reach of the Heihe River basin for the period 1984–2014, were simulated by SWAT, which was developed by the United States Department of Agriculture–Agricultural Research Services (USDA–ARS). The model parameters were calibrated using the remotely-sensed ET data on a monthly timescale for the period 2000–2013. Annual ET simulations for the region were produced.

### SWAT model

The SWAT model is hydrologic response unit-based (HRU). The basic calculation unit (HRU) is formed by dividing the watershed into 31 sub-basins based on the digital elevation model (DEM) and the distribution of stream channels, and further subdividing into 327 HRUs based on soil and land use characteristics. In each HRU, the water balance equation is used to describe the land surface hydrological cycle, and the responses of each HRU in terms of water, sediment, and nutrient are computed individually. The components are aggregated at the sub-basin level and routed to the catchment outlet through the river channel network. Hydrological response variables such as potential evapotranspiration and actual evapotranspiration, and other variables, can also be obtained during the model calculation process. A more detailed description of the SWAT model is given by Arnold *et al*.^8, 21, 22^, Neitsch *et al*.^23, 24^, and Gassman *et al*.^9^.

In the SWAT model, calculation of evapotranspiration requires parameters for vegetation canopy interception evaporation, soil evaporation, and crop transpiration. Each component needs to be calculated on the basis of potential evapotranspiration (PET) and observed environmental factors. The calculation order is PET, canopy interception evaporation, potential soil evaporation, and crop transpiration. The potential soil evaporation and crop transpiration values are distributed to each soil layer. Finally the soil evaporation and crop transpiration are calculated according to the soil moisture content of each layer. In this study, PET was simulated using the Penman–Monteith method^25^. The meteorological data required for this method include air temperature, solar radiation, relative humidity, and wind speed. Air resistance and canopy resistance are estimated based on the crop height and the leaf area index (LAI). Potential soil evaporation is determined by PET of crop and soil coverage. When the soil moisture content is lower than the field capacity, the actual soil evaporation will be limited by the soil moisture content and be exponentially related to the soil layer thickness. The crop transpiration calculation is similar to the soil evaporation calculation, and is exponentially related to crop depth.

### Model inputs and model setup

The climate data at eight meteorological stations (Dingxin, Jiuquan, Gaotai, Zhangye, Shandan, Tuole, Yeniugou, and Qilian) for the period 1982–2014 were used in the SWAT model. Data included daily precipitation, the minimum and maximum air temperature, wind speed, relative humidity, and global solar radiation. The main crop planted in the region is corn, which accounts for 77.19% of the crop acreage. Because there was little crop management data for other crops, this study considers only the agricultural management practices for corn, which include irrigation, fertilization, planting, and harvesting. The irrigation and fertilization schedule of each sub-basin was set to be the same as that of the irrigation zone where the center of the sub-basin was located. The auto-fertilization option was used to apply phosphorus fertilizer without consideration of phosphorus stress.

### Sensitivity analysis, model calibration and validation

Sensitivity analysis and calibration were performed on each sub-basin. The river flow is greatly affected by human activity: river water is mainly used for crop consumption (ET). Irrigation results in ET, so the ET data were selected as the objectives for parameter estimation. ET data for the period 2000–2013, which were derived from the ETWatch model^26^ with a resolution of 1 km, were obtained from the Data Management Center of the Heihe Research Program (http://westdc.westgis.ac.cn/). The data were aggregated into the SWAT model at the sub-basin scale as the observed data. Eddy covariance (EC) observations from two ground observation stations in the Heihe River basin (the Arou station and the Yingke station) were used for the verification of remotely-sensed ET data, and the observed daily values of ET for 2008 were compared with ET values estimated by the ETWatch model. The Yingke station is in an agricultural oasis typical of the basin. The terrain is very flat and the main crop is corn. For the Yingke oasis plain area, the coefficient of determination (*R* ^2^) is 0.9249, the RMSE is 0.5288 mm/d, and the difference between RMSE and the mean absolute error (MAE) is <40% of MAE. In addition, the index of agreement, *d*, is 0.978, which also suggests a good match between simulated and observed values^27^. The monthly ET value of the 31 sub-basins was first calculated using the Zonal toolset of ArcGIS. A two-year period was used to warm up the SWAT model for both the sensitivity analysis and calibration.

The Latin hypercube one-factor-at-a-time (LH-OAT)^28^ method was used for sensitivity analysis. Based on the literature of the sensitivity analysis of SWAT model parameters^28–30^, and with due consideration of the situation in the agricultural region, 15 parameters were selected for sensitivity analysis. The sequential uncertainty fitting algorithm version 2 (SUFI-2)^31, 32^, which is linked to the SWAT model by the SWAT-CUP program, was used for parameter calibration and validation. SUFI-2 gives a selected parameter a large initial range of values, sets a uniform distribution, and can deal with a large number of parameters through Latin hypercube sampling^31^. Following the recommendations of Abbaspour *et al*.^32^, the number of Latin hypercube samples was 500. In SUFI-2, the parameter uncertainty (i.e. the range of the parameter) is assumed to account for all sources of uncertainties (such as uncertainty in driving variables, in the conceptual model, in model parameters, or in observed data). The parameter uncertainty causes the uncertainty in the output which can be quantified by the 95% prediction uncertainty (95PPU). This is calculated at the 2.5% and 97.5% levels of the cumulative distribution of an output variable generated by the propagation of the parameter uncertainties using Latin hypercube sampling. Two indices are used to quantify the strength of the calibration–uncertainty performance: P-factor, which is the percentage of measured data bracketed by the 95PPU band, and R-factor, which is the average width of the band divided by the standard deviation of the corresponding measured variable. Ideally, we would like to bracket most of the measured data (plus their uncertainties) within the 95PPU band (P-factor → 1) while having the narrowest band (R-factor → 0)^32^. In order to compare the observed and simulated monthly ET, we used a slightly modified version of the efficiency criterion Φ^33^.

1 $${\rm{\Phi }}=\{\begin{array}{cc}|b|{R}^{2} & {\rm{for}}\,|b|\le 1\\ {|b|}^{-1}{R}^{2} & {\rm{for}}\,|b| > 1\end{array}$$

where *R* ^2^ is the coefficient of determination between the observed and simulated signals, and *b* is the slope of the regression line.

We also used the Nash–Sutcliffe (NS) coefficient, coefficient of determination (*R* ^2^), relative bias (RB), root mean square error (RMSE) and three other indices representing the less-often-employed but more interpretable class of measures based on sums of error magnitudes (E, E_1_, and d_r_)^34^ to determine the performance of the model.

2 $$NS=1-\frac{\sum _{i=1}^{n}{(E{T}_{o,i}-E{T}_{s,i})}^{2}}{\sum _{i=1}^{n}{(E{T}_{o,i}-\overline{E{T}_{o}})}^{2}}$$

3 $${R}^{2}=\frac{{[\sum _{i=1}^{n}(E{T}_{o,i}-\overline{E{T}_{o}})(E{T}_{s,i}-\overline{E{T}_{s}})]}^{2}}{\sum _{i=1}^{n}{(E{T}_{o,i}-\overline{E{T}_{o}})}^{2}\cdot \sum _{i=1}^{n}{(E{T}_{s,i}-\overline{E{T}_{s}})}^{2}}$$

4 $$RB=\frac{\sum _{i=1}^{n}(E{T}_{s,i}-E{T}_{o,i})}{\sum _{i=1}^{n}E{T}_{o,i}}$$

5 $$RMSE=\sqrt{\frac{1}{{\rm{n}}}{\sum _{i=1}^{{\rm{n}}}(E{T}_{s,i}-E{T}_{o,i})}^{2}}$$

6 $$E=1-\frac{MSE}{{{s}_{o,i}}^{2}}=1-\frac{\frac{1}{n}\sum _{i=1}^{n}{(E{T}_{s,i}-E{T}_{o,i})}^{2}}{{{s}_{o,i}}^{2}}$$

7 $${E}_{1}=1-\frac{MAE}{MAD}=1-\frac{\frac{1}{n}\sum _{i=1}^{n}|E{T}_{s,i}-E{T}_{o,i}|}{\frac{1}{n}\sum _{i=1}^{n}|E{T}_{o,i}-\overline{E{T}_{o}}|}$$

8 $${d}_{r}=\{\begin{array}{c}1-\frac{MAE}{2\cdot MAD}=1-\frac{\frac{1}{n}\sum _{i=1}^{n}|E{T}_{s,i}-E{T}_{o,i}|}{2\cdot \frac{1}{n}\sum _{i=1}^{n}|E{T}_{o,i}-\overline{E{T}_{o}}|}\,{\rm{when}}\,\frac{1}{n}\sum _{i=1}^{n}|E{T}_{s,i}-E{T}_{o,i}|\le 2\cdot \frac{1}{n}\sum _{i=1}^{n}|E{T}_{o,i}-\overline{E{T}_{o}}|\\ \frac{2\cdot MAD}{MAE}-1=\frac{2\cdot \frac{1}{n}\sum _{i=1}^{n}|E{T}_{o,i}-\overline{E{T}_{o}}|}{\frac{1}{n}\sum _{i=1}^{n}|E{T}_{s,i}-E{T}_{o,i}|}-1\,{\rm{when}}\,\frac{1}{n}\sum _{i=1}^{n}|E{T}_{s,i}-E{T}_{o,i}| > 2\cdot \frac{1}{n}\sum _{i=1}^{n}|E{T}_{o,i}-\overline{E{T}_{o}}|\end{array}$$

where *ET*_*o,i*_ represents the measured *ET* value; *ET*_*s,i*_ represents the simulated *ET* value; $\overline{E{T}_{o}}$ represents the mean of the measured *ET* value; $\overline{E{T}_{s}}$ represents the mean of the simulated *ET* value; ${{s}_{o,i}}^{2}$ represents the variance of the simulated *ET* value; and *n* is the time series length.

### The contribution rate of ET change

Factor analysis was used to determine the contribution rates of human agricultural management activity and of climate change to change in ET. The purpose of factor analysis is to combine interrelated variables into a few factors to reproduce the relationships between the original variables and the factors. Variables can also be classified according to different factors. Factor analysis is a statistical method of dimension reduction in multivariate analysis. The mathematical model of factor analysis is represented by a matrix^35^:

9 $${X}_{p\times 1}={A}_{p\times k}{F}_{k\times 1}+{\varepsilon }_{p\times 1}$$

where $X=({X}_{1},{X}_{2},\mathrm{...},{X}_{p})$ is a P-dimensional stochastic vector composed of the observed *p* indicators; $F=({F}_{1},{F}_{2},\mathrm{...},{F}_{k})$, (*k* < *p*), is an unobservable vector and the common factor of *X*; and ${\varepsilon }_{i}(i=1,2,\ldots ,\,p)$ is a unique independent factor called the special factor which accounts, for each *X*_*i*_, for any errors.

Since the fluctuation of *X* is the same as that of *X* + *b* (*b* is a constant vector), it may be assumed that *E*(*X*) = 0. The model contains a large number of unobservable quantities, which cannot be directly determined. In order to examine the covariance relation, it is assumed that the components are not related, that the variance is 1, and that *F* and *ε* are independent of each other. The results of the factor analysis are represented by the common factor *F*_*j*_, the factor load of each variable *a*_*ij*_, the variable commonality ${{h}_{i}}^{2}$, and the factor contribution rate. The statistical significance of *a*_*ij*_ is that it is the correlation coefficient between the *i* variable and the *j* common factor, which means the load of the *i* variable on the *j* common factor. The variable commonality ${{h}_{i}}^{2}$ describes the contributions of all the common factors to the total variance of the variable *X*_*i*_, and the closer the value of ${{h}_{i}}^{2}$ to 1, the more the original information of the variable is explained by the common factor. When calculating the relative factor contribution rate, the factor load matrix of the top *k* factors, *A*_*p*×*k*_, is first to be calculated:

10 $${A}_{p\times k}=[\begin{array}{c}{a}_{11},{a}_{12},\mathrm{...},{a}_{1k}\\ {a}_{21},{a}_{22},\mathrm{...},{a}_{2k}\\ {a}_{p1},{a}_{p2},\mathrm{...},{a}_{pk}\end{array}]=[\begin{array}{c}{u}_{11}\sqrt{{\lambda }_{1}},{u}_{12}\sqrt{{\lambda }_{2}},\mathrm{...},{u}_{1k}\sqrt{{\lambda }_{k}}\\ {u}_{21}\sqrt{{\lambda }_{1}},{u}_{22}\sqrt{{\lambda }_{2}},\mathrm{...},{u}_{2k}\sqrt{{\lambda }_{k}}\\ {u}_{p1}\sqrt{{\lambda }_{1}},{u}_{p2}\sqrt{{\lambda }_{2}},\mathrm{...},{u}_{pk}\sqrt{{\lambda }_{k}}\end{array}]$$

where ${\lambda }_{1}\ge {\lambda }_{2}\ge {\lambda }_{3}\ge \mathrm{...}\ge {\lambda }_{k}$ are the first *k* characteristic values of the covariance matrix *R* of *X*_*p*×*l*_, and ${u}_{1},{u}_{2},\mathrm{...},{u}_{k}$ are the corresponding standard orthogonal eigenvectors. Two methods are used for determining *k*: according to the size of the eigenvalue, generally take an eigenvalue greater than 1; according to the cumulative variance contribution of the factor, which generally should be more than 80%. Then the variance contribution of the common factor *F*_*k*×*l*_ is obtained by calculating the sum of the squares of the elements in the *j* column of the factor load matrix *A*_*p*×*k*_:

11 $${S}_{j}=\sum _{i=1}^{p}{{a}_{ij}}^{2},j=1,2,\mathrm{...},\,p$$

which is the sum of squares of the elements in the factor loading matrix and represents the sum of the variance contributions of the same common factor *F*_*j*_ to the variables. It is an index of the relative importance of common factors. In addition, it can calculate the cumulative variance contribution rate of the former *k* common factors:

12 $$Q=\frac{\sum _{i=1}^{k}{\lambda }_{i}}{\sum _{i=1}^{l}{\lambda }_{i}}$$

It is generally believed that the cumulative contribution rate of the common factors is more than 80%, and that the common factors obtained can explain all the original information.

The long-term changes in ET result from the interactions between human-controlled agricultural production factors, irrigation factors, and natural change factors. These interactions embrace complex mechanisms and processes for change. Five meteorological factors (precipitation, average temperature, solar radiation, wind speed, and relative humidity) were selected to represent climate change factors. We also selected the following factors to represent human activity that effects change in ET per unit area: the ratio of the area of grain crops to the area of cash crops; the ratio of the irrigated area to the cultivated area; irrigation quota; plastic film mulch usage per unit area; and fertilizer usage per unit area. The following human activities that influence change in total ET in the region were selected: the ratio of the area of grain crops to the area of cash crops; the area under cultivation; the area that was irrigated; the irrigation quota; total fertilizer usage; and total plastic film mulch usage. Daily climate data were obtained from the China Meteorological Data Sharing Service System (http://data.cma.cn), and the remaining statistical data were obtained from the China Economic and Social Development Statistics Database (http://tongji.cnki.net/kns55/index.aspx), the Gansu Development Yearbook and the Gansu Rural Yearbook. Correlation analysis was first performed to determine the main driving factors that influenced change in ET. The normalized method was then used to standardize the main driving factors, using the following equation:

13 $${X}_{j}^{\text{'}}=({X}_{j}-{\bar{X}}_{j})/S{D}_{j}$$

where *X*_*j*_ is the indicator value of the *j* factor, $\mathop{{X}_{j}}\limits^{\_}$ is the average sample interval of the *j* index, and *SD*_*j*_ is the standard deviation of all sample data for the *j* index. The KMO test and Bartlett’s sphericity test^36, 37^ were used to ensure that the data were suitable for factor analysis. Finally the factor analysis was performed using SPSS.

## Results

### Model calibration and validation

Many SWAT model parameters are difficult to obtain through observation. Various parameters have differing effects on hydrological processes. Sensitivity analysis can identify the effects and importance of model parameters that can then be targeted for adjustment to improve the accuracy of the model. We analyzed the results from the hydrological processes calculation module of the SWAT model. The analysis provided a ranking of model parameters by order of importance. We finally selected the following 10 parameters to be calibrated: ESCO, CANMX, SOL_AWC, SOL_BD, EPCO, HVSTI, GW_REVAP, CN2, GSI and GWQMN (Table 1).

**Table 1:** Parameters used for SWAT model calibration.

Parameter^a^	Definition	Min	Max	Fitted value
v_ _ESCO.hru	Soil evaporation compensation factor	0	1	0.94
v_ _CANMX.hru	Maximum canopy storage(mm)	0	100	16.25
r_ _SOL_AWC.sol	Soil available water storage capacity(mm H_2_O/mm soil)	−0.3	0.3	−0.05
r_ _SOL_BD.sol	Moist bulk density(g/cm^3^)	−0.3	0.3	0.24
v_ _EPCO.hru	Plant uptake compensation factor	0	1	0.10
v_ _HVSTI.crop.dat	Harvest index	0.01	1.0	0.52
v_ _GW_REVAP.gw	Groundwater “revap” coefficient	0.02	0.2	0.05
r_ _CN2.mgt	SCS runoff curve number for moisture condition II	−0.3	0.3	−0.21
v_ _GSI.crop.dat	Maximum stomatal conductance (m/s)	0	5	1.77
v_ _GWQMN.gw	Threshold depth of water in the shallow aquifer required for return flow to occur (mm)	0	5000	800

^a^v_ _: parameter value is replaced by given value or absolute change; r_ _: parameter value is multiplied by (1 + a given value) or relative change.

The four evaluation indices (P-factor, R-factor, NS and Φ) were optimized by repeatedly adjusting the range of each parameter until we obtained a steady parameter value. Table 1 lists the calibrated parameters and their descriptions, and their initial and optimal values. The smaller the soil evaporation compensation factor (ESCO), the more water can be absorbed from the lower level soil for soil evaporation. Arnold *et al*.^38^ suggest that the value of ESCO should be varied between 0.75 and 1.0, and we used the value 0.94. This relatively high ESCO value indicates that soil evaporation has a weak influence on the whole evaporation process because of the high vegetation coverage, high plastic film mulch coverage, and low air temperature in the Heihe agricultural region. The maximum canopy storage (CANMX) has a significant effect on hydrological processes, especially for evapotranspiration in arid areas. The value of CANMX for farmland in this study was 16.25 (mm). The plant uptake compensation factor (EPCO) has an important effect on the ET simulation. When the EPCO value is close to 1, the crop can extract more water from the lower soil layer; when it is closer to 0, water absorption from the soil is limited. The calibrated value of EPCO was 0.1, mainly because local soil is very porous and there is little capillary water movement. The harvest index (HVSTI) and maximum stomatal conductance (GSI) were calibrated to 0.52 and 1.77 m/s, respectively, as given by Jiang *et al*.^39^. The SCS runoff curve number (CN2) was eventually reduced by 0.21 from the initial values, similar to the values given in Li *et al*.^40^. The soil-related sensitivity parameters (SOL_AWC, SOL_BD) were reduced by 0.05 and 0.24, respectively. The parameters GW_REVAP and GWQMN, which are correlated with groundwater flow, were calibrated to 0.05 and 800 mm, respectively.

**Figure 2:**
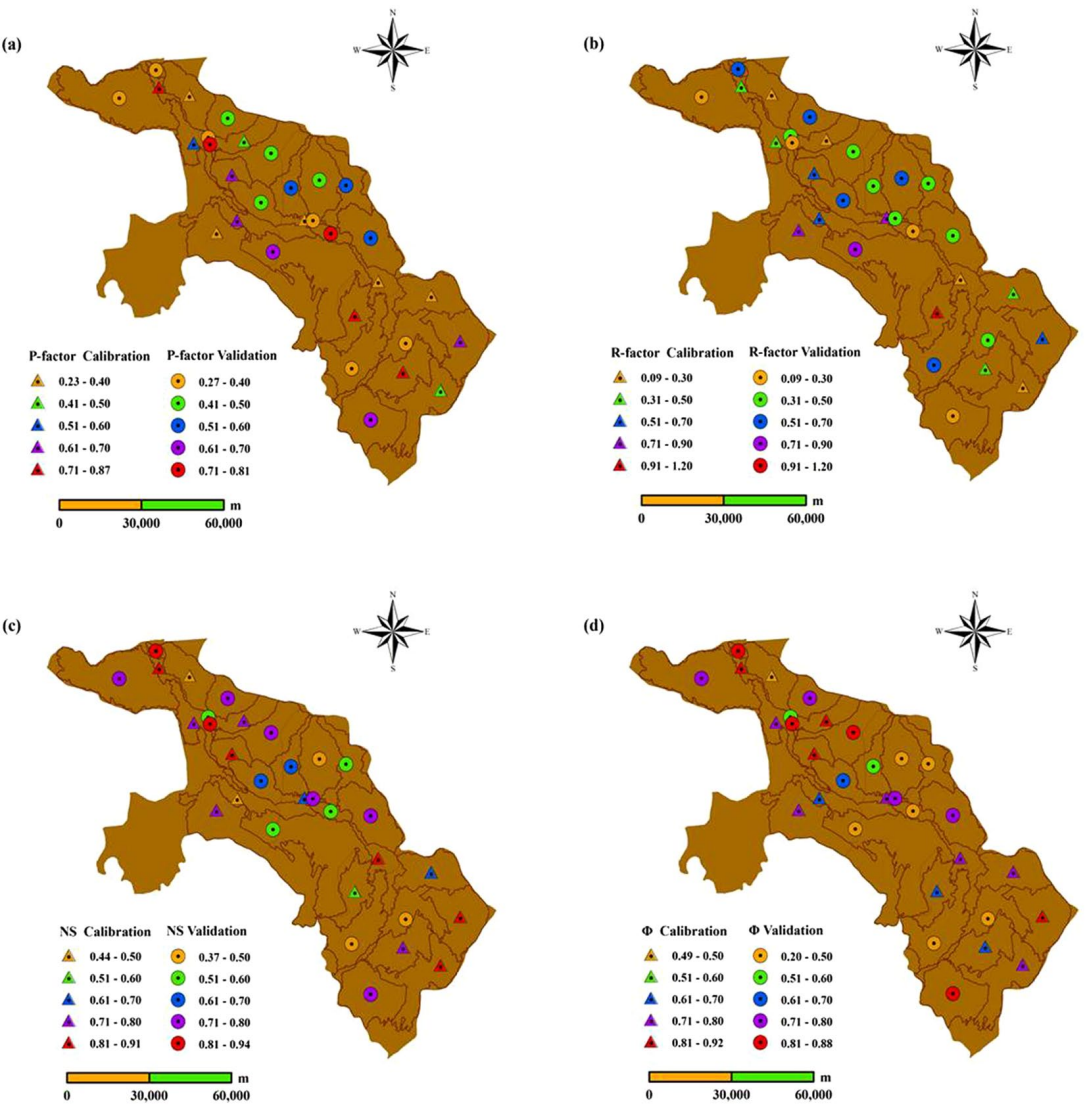
The P-factor, R-factor, NS coefficient and Φ for model calibration and validation at 31 sub-basins. The maps were created using ArcGIS10.0 software provided by the Environmental Systems Research Institute (http://www.esri.com).

As suggested in Sun *et al*.^41^, we selected 14 of the 31 sub-basins in the Heihe agricultural region, which collectively contain all land use and soil types in the region, combined them with the ET data, and used them as the objectives for parameter calibration. The remaining 17 sub-basins were selected for validation. As shown in Fig. 2, the P-factor was >0.5 for 50% of the calibrated sub-basins and for 41.2% of the validated sub-basins. The R-factor was relatively small, its value was always <1.0. The NS coefficient value was >0.6 in 78.6% of the calibrated sub-basins and in 58.8% of the validated sub-basins. The model also calculated the efficiency criteria of the best simulation. The Φ value was >0.7 in 71.4% of the calibrated sub-basins and in 52.9% of the validated sub-basins. Overall, we concluded that the calibrated SWAT model accurately represents the study area and that the results are acceptable for use, particularly in the agricultural area.

**Figure 3:**
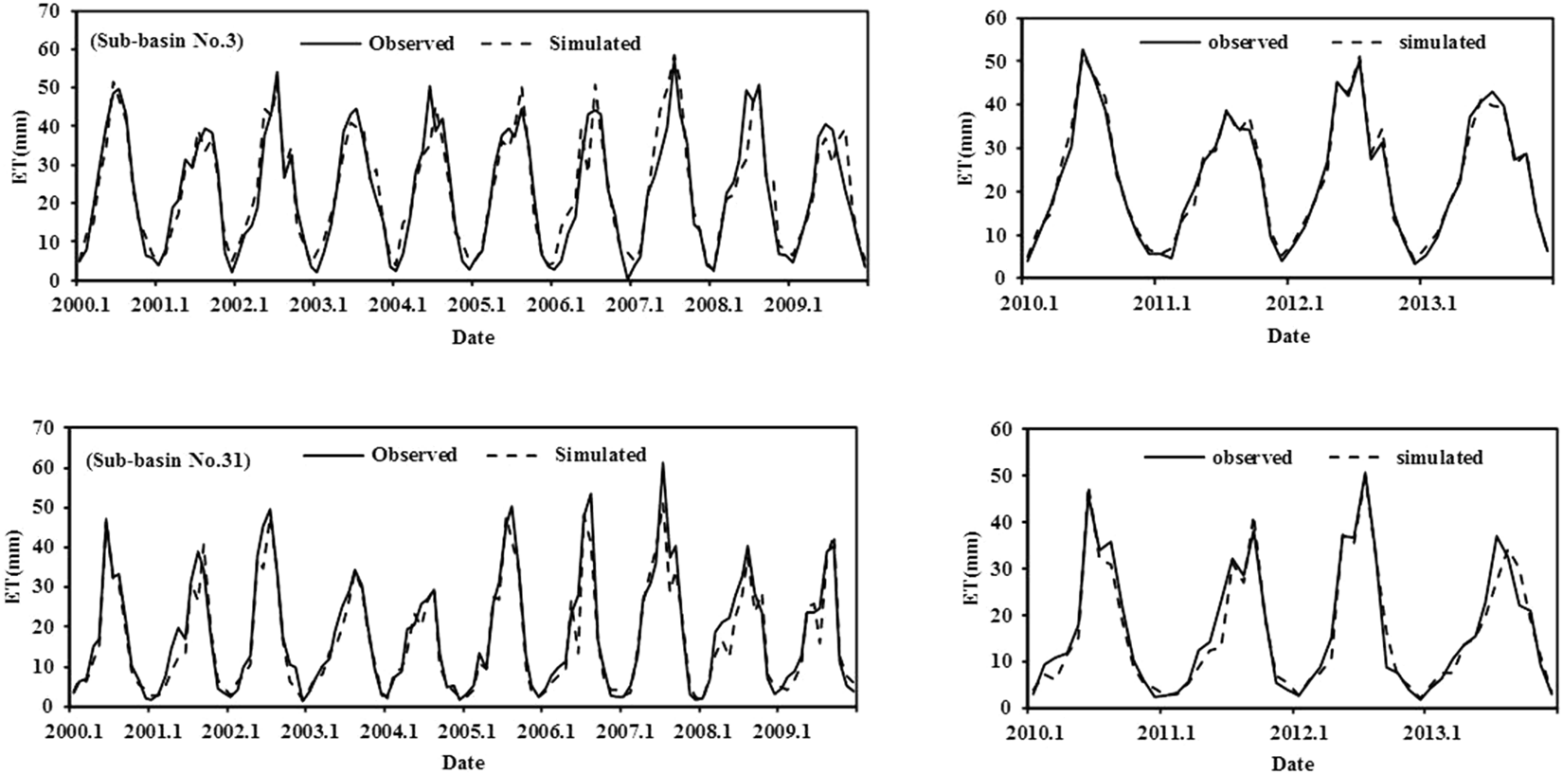
Simulated and observed monthly evapotranspiration (ET) at two typical sub-basins (numbers 3 and 31) for the calibration period (2000–2009) and the validation period (2010–2013).

The results were further evaluated by comparisons of simulated ET and observed ET in typical sub-basins, which are predominantly bare soil (sub-basin 3) and cultivated land (sub-basin 31). Figure 3 and Table 2 show that the simulation agreed well with the observed values for two typical sub-basins, and the statistical values of the indicators demonstrated that the model performed well. The NS and Φ values were mostly ≥0.7, RMSE was in the range 1.72–4.95 mm, RB was in the range −0.08 to +0.02, and the indicators E, E_1_, and d_r_ were simultaneously close to 1. These values also indicate that the calibrated SWAT model reasonably well simulates ET in the area. The ETWatch platform uses daily net radiation, soil moisture, and wind speed, which have been shown to be the most important factors of surface resistance, to estimate daily surface resistance. ETWatch combines these factors with meteorological data using the Penman–Monteith equation to estimate daily ET. However, ET calculation in SWAT is indirect since it does not use meteorological factors to directly calculate ET. So we can find that the method of calculating ET is independent between these two models, and thus we conclude that the SWAT model provides reliable data for the analysis of changes in ET and permits accurate identification of the factors that drive the changes.

**Table 2:** ET simulation results at two typical sub-basins (numbers 3 and 31) for the calibration and validation period.

sub-basin	Calibration period (2000–2009)							Validation period (2010–2013)						
	NS	Φ	RB	RMSE (mm)	E	E_1_	d_r_	NS	Φ	RB	RMSE (mm)	E	E_1_	d_r_
No. 3	0.71	0.79	0.02	4.95	0.89	0.73	0.87	0.70	0.80	0.02	1.72	0.98	0.89	0.94
No. 31	0.72	0.78	−0.08	3.95	0.92	0.77	0.89	0.69	0.71	−0.04	3.19	0.94	0.81	0.91

## Discussion

### Driving factors for changes in total ET and ET per unit area

**Figure 4:**
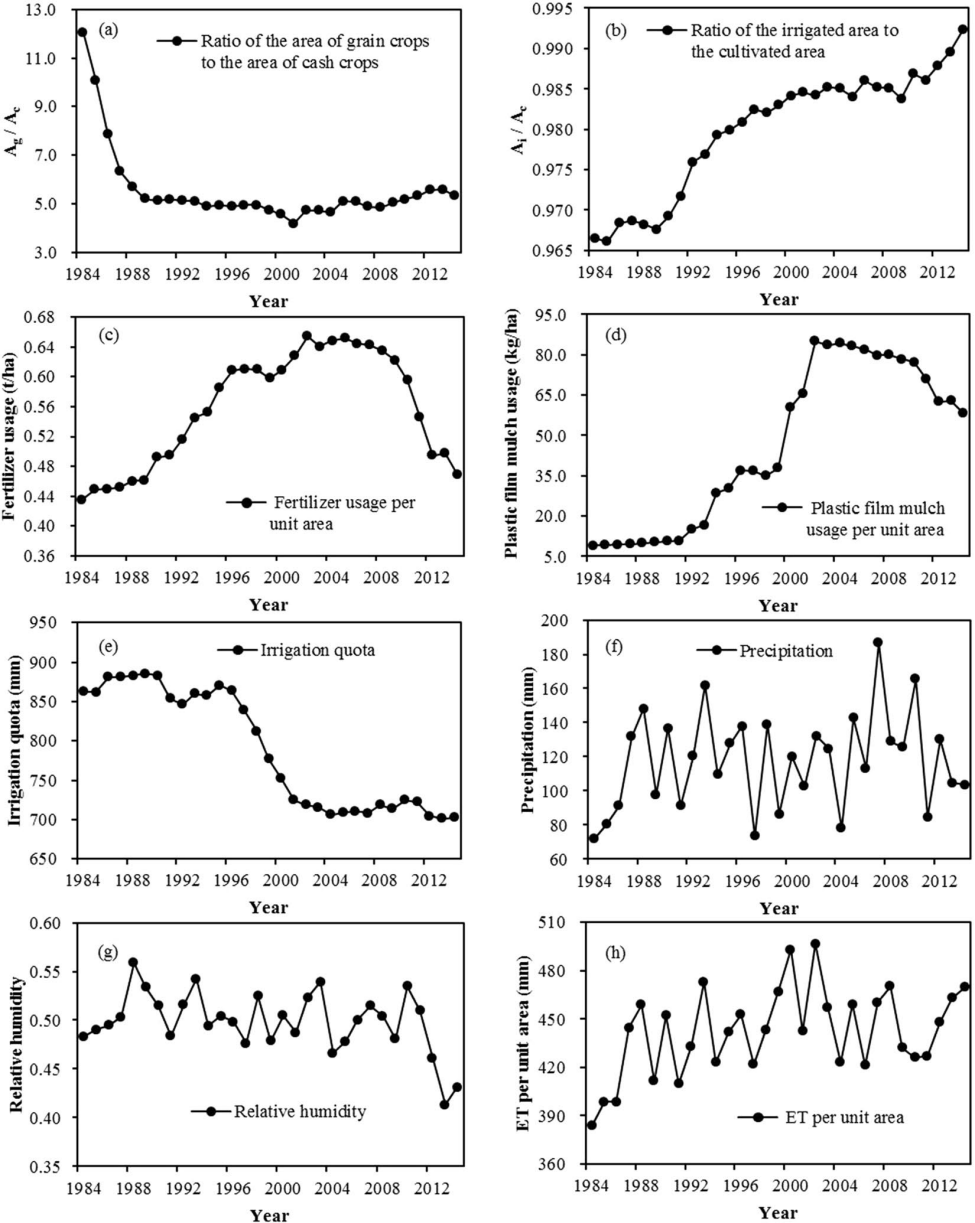
The change in average ET per unit area for the entire agricultural region and the main driving factors.

**Figure 5:**
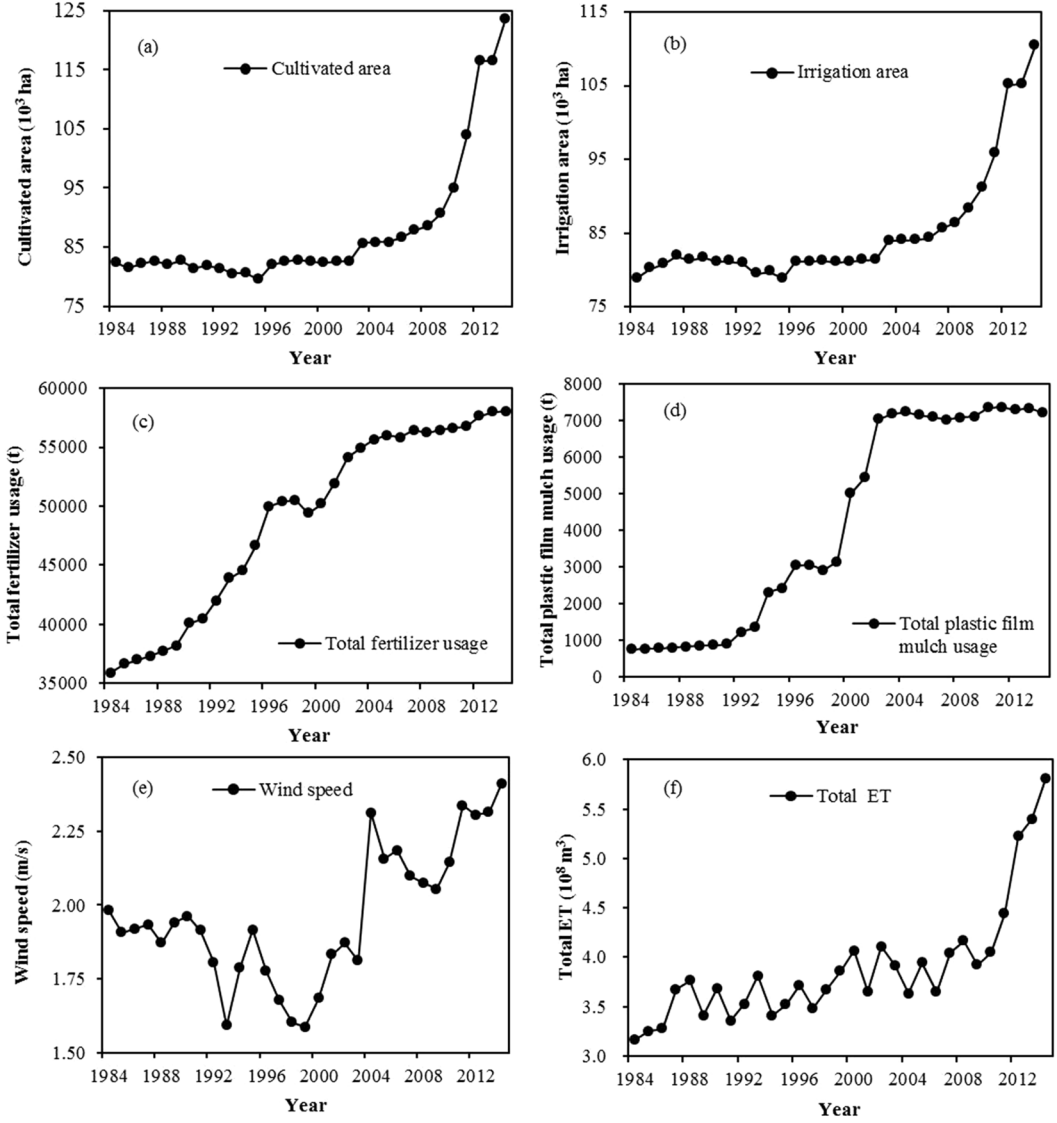
The change in average total ET for the entire agricultural region and the main driving factors.

The average annual ET per unit area derived from the SWAT model in the Heihe agricultural region was 442.1 mm for the period 1984–2014. The maximum value was 496.62 mm, in 2002, and the minimum value was 383.81 mm, in 1984, as shown in Figs 4 and 5. The ratio of the maximum ET value to the minimum ET value was 1.29, and the coefficient of variation was 0.06. These two values show that the inter-annual fluctuation of ET was relatively small. There was a slight increasing trend of ET per unit area during the 31-year period, with the trend coefficient |*r*| = 0.1834 < *r*_0.05_. ET per unit area represents water consumption intensity. It is determined mainly by local meteorological factors and by irrigation water usage (the irrigation quota). ET per unit area is also influenced by other factors, such as the crop planting structure and water-saving irrigation measures. These influences are dynamic and change from year to year, so an interannual fluctuation in ET per unit area is to be expected. The average annual total ET was 3.89 × 10^8^ m^3^. A significant increasing trend in total ET was found, with the trend coefficient |*r*| = 0.6004 > *r*_0.05_. Total ET represents the total amount of water consumption, and it is directly related to the area under cultivation. The increase in total ET was more noticeable after 2010. This increase was mainly due to the rapid expansion in the area of land under cultivation after 2010^42^.

Table 3 gives the values of the coefficients of correlation between ET per unit area and its driving factors, and between total ET and its driving factors. The results show that ET per unit area was positively correlated with the following: the ratio of the area of grain crops to the area of cash crops; the ratio of the irrigated area to the cultivated area; fertilizer usage per unit area; plastic film mulch usage per unit area; and precipitation. ET per unit area was negatively correlated with the irrigation quota and with relative humidity. There was no significant correlation with solar radiation, average temperature, or wind speed. Total ET was positively correlated with the following: the ratio of the area of grain crops to the area of cash crops; cultivated area; irrigation area; total fertilizer usage; total plastic film mulch usage; and wind speed. Total ET was negatively correlated with the irrigation quota and with relative humidity. There was no significant correlation between total ET and average temperature, precipitation, or solar radiation.

**Table 3:** Correlation coefficients (r) between ET per unit area, total ET and driving factors.

ET per unit area		Total ET	
Factors	r	Factors	r
Ratio of the area of grain crops to the area of cash crops	0.693**	Irrigation area	0.935**
Relative humidity	−0.622**	Cultivated area	0.929**
Ratio of the irrigated area to the cultivated area	0.586**	Total fertilizer usage	0.660**
Precipitation	0.538**	Total plastic film mulch usage	0.646**
Plastic film mulch usage per unit area	0.526*	Irrigation quota	−0.640**
Irrigation quota	−0.519*	Wind speed	0.591**
Fertilizer usage per unit area	0.511*	Ratio of the area of grain crops to the area of cash crops	0.574**
Wind speed	0.109	Relative humidity	−0.511**
Solar radiation	0.077	Precipitation	0.166
Average temperature	0.056	Solar radiation	0.075
		Average temperature	0.058

*Denotes correlation is significant at 0.05 level; **denotes correlation is significant at 0.01 level.

The increase in the area of land under cultivation, and the increase in the area of land being irrigated, in conjunction with changes in planting practices (both the ratio of the area of grain crops to the area of cash crops and the area of grain crop cultivation increased), all led to an increase in ET. Other factors which contributed to the increase in ET include: the wide adoption of drip irrigation and other water-saving measures; the increased use of chemical fertilizers^43–45^; and the increased use of plastic film mulch. The use of drip irrigation and other water-saving measures decreased the amount of water used for irrigation and to some degree increased the effectiveness of fertilizer use. These measures promoted crop growth^46, 47^, and thus may have caused ET to increase accordingly, so ET was negatively correlated with the quantity of water used for irrigation between 1984 and 2014. There was a positive correlation between ET and the use of plastic film mulch. Plastic film mulch has been widely used in northern China for many years to modify the soil surface. It leads to an increase in topsoil temperature which promotes early growth, it maintains soil water content, and it promotes increased yield^48–50^. Although plastic film mulch reduces soil evaporation at the early stage of plant growth, ET increased during the overall growth period in northwest China^51^. Higher relative humidity can reduce the water vapour gradient between a plant leaf and the atmosphere, which lowers the water vapour diffusion rate, and thus reduces evaporation so that there is a decrease in ET. As shown in Figs 4 and 5, ET had an increasing trend, while relative humidity was decreasing. Thus ET has a negative correlation with relative humidity. Precipitation increases the soil moisture content, which increases the likelihood of evaporation. In addition, precipitation satisfies some of the crop water demand and so promotes crop growth. Crop growth leads to increased ET. Thus precipitation has some effect in increasing ET. As wind speed increases, the water vapour diffusion resistance decreases, which leads to increased ET. In a study of the sensitivity of ET to global climate change in the arid zone of Rajasthan, India^52^, when wind speed increased by 20%, ET increased by 7%; as humidity increased by 20%, ET decreased by 4.3%. Our study data show that the average temperature and solar radiation in the 31-year period were relatively stable in the study region, and thus had little effect on ET.

We selected the factors that have significant correlations with ET per unit area and those that have significant correlations with total ET, based on the preceding analysis, to calculate the contribution rate. The main driving factors of changes in ET per unit area and total ET are shown in Fig. 4 and Fig. 5.

### Contribution rates of driving factors for change in ET per unit area

Prior to undertaking factor analysis we used the Kaiser–Meyer–Olkin (KMO) test^36^ and Bartlett’s sphericity test^37^ to see whether the indicators of change in ET per unit area were suitable for factor analysis. The KMO test examines partial correlation between variables to show that there is no significant difference in the degree of correlation between variables. The KMO value was 0.743, which satisfied the minimal requirement of being >0.5^36^. Bartlett’s sphericity test was used to check whether the correlation array was a unit array, that is, whether the variables were independent. The results show that the Bartlett’s sphericity test value was 139.356, the degree of freedom was 36, and the P value was 0, which satisfies the requirement that P < 0.001. The unit matrix assumption was not established, and met the prerequisites for extracting common factors. Based on the two sets of test results, the data is suitable for factor analysis.

The results of the factor analysis are given in Tables 4 and 5. The results show that the first common factor is composed of the following: the ratio of the area of grain crops to the area of cash crops; the ratio of the irrigated area to the cultivated area; fertilizer usage per unit area; and plastic film mulch usage per unit area. This is the agronomy factor. The factor contribution rate of the agronomy factor is 43.24%. The second common factor, the irrigation factor, is composed of the irrigation quota, with a factor load 0.707 and contribution rate of 17.69%. The contribution rate of human agricultural activities to the increase in ET per unit area is the sum of the agronomy factor and the irrigation factor, 60.93%. The contribution rate of the third common factor, the climate factor, is 28.01%. The factor loads of precipitation and relative humidity are 0.695 and −0.764, respectively. The climate factor represents the contribution rate of climate change (measured as precipitation and relative humidity) to change in ET per unit area.

**Table 4:** Rotated component matrix in factor analysis of ET per unit area.

Items	Human activity		Climate change
	Agronomy factor	Irrigation factor	Climate factor
Ratio of the area of grain crops to the area of cash crops	0.906	−0.177	−0.006
Ratio of the irrigated area to the cultivated area	0.875	−0.098	−0.078
Fertilizer usage per unit area	0.879	−0.083	−0.015
Plastic film mulch usage per unit area	0.927	−0.138	−0.023
Irrigation quota	0.326	0.707	−0.108
Precipitation	0.231	−0.374	0.695
Relative humidity	0.531	−0.028	−0.764

**Table 5:** Total variance explained in factor analysis for ET per unit area and total ET.

Items	Component	Rotation Sums of Squared Loadings		
		Eigen value	Variance contribution (%)	Cumulative variance contribution (%)
ET per unit area	Agronomy factor	3.027	43.24	43.24
	Irrigation factor	1.592	17.69	60.93
	Climate factor	1.758	28.01	88.94
Total ET	Agronomy factor	3.489	43.61	43.61
	Irrigation factor	1.082	10.25	53.86
	Climate factor	2.854	35.68	89.54

The main driving factor of change in ET per unit area in the Heihe agricultural region is the agronomy factor. The irrigation factor (which reflects the level of water saving in the region) also has a significant influence on the change in ET per unit area. This result indicates that the influence of water saving measures on agricultural ET cannot be neglected. The climate factor also influences changes in ET. However, human agricultural management activity has a greater effect on change in ET per unit area than climate change has. The region studied typifies agricultural irrigation use because of limited regional water resources. Water used for irrigation is mainly consumed through ET. Agronomic measures, such as the use of chemical fertilizers, plastic film mulch, and water-saving irrigation, significantly promote the growth of crops^45–51^. Average temperature and solar radiation have been comparatively stable over the 31-year period. The relative contribution of human agricultural management activity to change in ET per unit area was much larger than that of the climate variables. Thus human activity is the main driving factor of change in ET per unit area.

### Contribution rates of driving factors to total ET change

The KMO value for the driving factors of change in total ET was 0.779, which satisfies the minimal requirement of being >0.5^36^. The Bartlett’s sphericity test value was 274.189, and the P value was <0.001. Both the KMO test and Bartlett’s sphericity test demonstrate the suitability of the data for factor analysis. The results of the factor analysis are given in Tables 5 and 6. The first common factor is composed of the following: the ratio of the area of grain crops to the area of cash crops; the total cultivated area; total fertilizer usage; and total plastic film mulch usage. The factor load values were 0.759, 0.941, 0.857, and 0.831, respectively. The first common factor is the agronomy factor, and its factor contribution rate is 43.61%; the second common factor, the irrigation factor, consists of the irrigation quota and the irrigation area. Its contribution rate is 10.25%. The contribution rate of human agricultural management activity to change in total ET is the sum of the contribution rates of the agronomy factor and the irrigation factor, 53.86%. The contribution rate of the third common factor, the climate factor, is 35.68%, and the factor load values of relative humidity and wind speed are −0.709 and 0.857, respectively.

**Table 6:** Rotated component matrix in factor analysis of total ET.

Items	Human activity		Climate change
	Agronomy factor	Irrigation factor	Climate factor
Ratio of the area of grain crops to the area of cash crops	0.759	−0.007	−0.128
Cultivated area	0.941	0.004	0.054
Total fertilizer usage	0.857	0.011	−0.017
Total plastic film mulch usage	0.831	0.325	0.005
Irrigation quota	−0.298	0.655	−0.117
Irrigation area	0.003	0.947	0.082
Relative humidity	0.592	−0.026	−0.709
Wind speed	0.092	−0.193	0.857

The human activity factors were the main driving force of change in total ET. This result is consistent with the identification of the driving forces of change in ET per unit area. The relative contribution of the climate factor to change in total ET (relative humidity and wind speed) was 7.67% greater than its influence on the change in ET per unit area. This result indicates that climate change is a more significant contributor to change in total ET than to change on ET per unit area. The contribution of human activity to changes in total ET still predominates, which is consistent with the corresponding result for change in ET per unit area. The area under cultivation shows an increasing trend in ET, which is more evident after 2010, although there is limited precipitation in the region. Irrigation is necessary to supply water to crops, and the cultivated area being irrigated also showed an increasing trend in ET. Increased use of chemical fertilizer and of plastic film mulch promotes crop growth, so these two crop management activities (constituents of the agronomy factor) become the main driving forces which influence the change in total ET. Some meteorological influences clearly changed inter-annually (wind speed, relative humidity). Other meteorological influences which also provide energy for ET to occur (average temperature, solar radiation) showed little inter-annual change. We conclude that the contribution of human agricultural management activity to total ET change in the 31-year period was greater than that of climate change.

### Uncertainty analysis

Overall, the results suggest that both climatic and human activity factors are influential as driving mechanisms of ET change in the Heihe agricultural region. However, there are still some uncertainties and more in-depth analysis is necessary. For example, the SWAT model did not take into account any effects of land use change during the period of ET simulation. Only one land use map was used, because limited data was available, although the area of land used for cultivation in the region has been increasing since 2000^42^. There may be data errors, such as inaccurate irrigation schedules in the statistical yearbooks. Lack of accurate irrigation data may mean that some sub-basins (those in which mainly vegetables, cotton and other crops are planted) are not properly represented in the model. A too-low irrigation quantity will cause ET to be underestimated. This error in ET estimation will have an impact on the subsequent analysis and selection of the main driving factors, which will in turn further affect the accuracy of the contribution rates.

Any inaccuracy in the driving variables will also affect the estimation of the influence of the factors. For example, there are few meteorological stations within and around the Heihe agricultural region. Thus data for climate variables in any sub-basin will have some degree of inaccuracy due to the distance from the point of data collection. Inaccurate data may affect the results of correlation analysis and subsequent calculations of factor influence and contribution rates. The data for the agronomic and irrigation factors are obtained from the statistical yearbooks. If there are errors in the yearbooks (however caused), the selected driving factors will not be realistic. The resulting contribution rate calculations will be inaccurate.

Calculation of the contribution rates of the driving factors of change in ET involves uncertainty. For example, the driving factors of change in ET per unit area are not consistent with those for change in total ET. A limitation of factor analysis is that the driving factors are not independent, they may interact with each other and changes in one factor can cause changes in other factors. ET is a very complex process but this study analyzed only the major factors which influence it (human activity and climate change). Other influential factors, such as changes in crop varieties or in groundwater extraction, which are difficult to quantify and require a more comprehensive model, were not considered. The results would probably have been different when we included these elements in the factor analysis in order to calculate their contribution rate.

Global warming and human agricultural activities are both changing the agricultural environment in the Heihe agricultural region. Quantifying these changes will lead to a better understanding of the behaviour of ET, and the factors that influence it, within the regional water cycle. Increased understanding of ET will enable us to develop better decision support systems to manage water consumption in response to these changes. This study, despite the uncertainties we have identified, provides valuable reference information to facilitate the creation of appropriate management measures. Water is a scarce agricultural resource in the Heihe River basin so we must promote water usage efficiency. Knowledge of the factors which influence ET and how they do so will enable us to maintain the productivity of the agricultural ecosystem.

## Conclusions

Comprehensive analysis of recent changes in land ET and of their contributions in terms of variable climatic and human factors is a challenging task due to the complex interactions between the factors. Using remotely-sensed ET products and SWAT modeling, this study analyzed changes in ET in the Heihe agricultural region over a 31-year period, and correlation analysis identified the dominant factors influencing ET per unit area and total ET. Factor analysis was used to quantify the contribution rate of each dominant factor to ET per unit area and to total ET. In the study area, the contribution rate of human activities, including the agronomic and irrigation factors, to increased ET per unit area and to increased total ET in the 31-year period was significantly larger than the respective contribution rates of climate change. There are some uncertainties in the results, and it is necessary to perform further analysis using more accurate data and more robust methods, but these results still provide valuable reference information to guide the improvement of agricultural water management.

To reduce the demands of agricultural water usage in the Heihe River basin, and to ensure the sustainability of water resources in this arid region of northwest China, it is necessary to further rationalize the scale of agricultural production, to control the use of both plastic film mulch and fertilizer, and to implement water-saving measures. This conclusion shows the need to establish an effective agricultural water-saving regime by considering both the region’s social and environmental conditions and changes in agricultural water consumption caused by climate change.
